# Deep-learning-based washout classification for decision support in contrast-enhanced ultrasound examinations of the liver

**DOI:** 10.1117/1.JMI.12.4.044502

**Published:** 2025-07-22

**Authors:** Hannah Strohm, Sven Rothlübbers, Jürgen Jenne, Dirk-André Clevert, Thomas Fischer, Niklas Hitschrich, Bernhard Mumm, Paul Spiesecke, Matthias Günther

**Affiliations:** a Fraunhofer Institute for Digital Medicine MEVIS, Bremen, Germany; b Ludwig-Maximilians-University Munich, Department of Radiology, Munich, Germany; c Charité-Universitätsmedizin Berlin, Interdisciplinary Ultrasound Center, Department of Radiology, Berlin, Germany; d TOMTEC Imaging Systems, Unterschleißheim, Germany; e University of Bremen, Bremen, Germany

**Keywords:** contrast-enhanced ultrasound, deep learning, liver lesions, time intensity curves

## Abstract

**Purpose:**

Contrast-enhanced ultrasound (CEUS) is a reliable tool to diagnose focal liver lesions, which appear ambiguous in normal B-mode ultrasound. However, interpretation of the dynamic contrast sequences can be challenging, hindering the widespread application of CEUS. We investigate the use of a deep-learning-based image classifier for determining the diagnosis-relevant feature washout from CEUS acquisitions.

**Approach:**

We introduce a data representation, which is agnostic to data heterogeneity regarding lesion size, subtype, and length of the sequences. Then, an image-based classifier is exploited for washout classification. Strategies to cope with sparse annotations and motion are systematically evaluated, as well as the potential benefits of using a perfusion model to cover missing time points.

**Results:**

Results indicate decent performance comparable to studies found in the literature, with a maximum balanced accuracy of 84.0% on the validation and 82.0% on the test set. Correlation-based frame selection yielded improvements in classification performance, whereas further motion compensation did not show any benefit in the conducted experiments.

**Conclusions:**

It is shown that deep-learning-based washout classification is feasible in principle. It offers a simple form of interpretability compared with benign versus malignant classifications. The concept of classifying individual features instead of the diagnosis itself could be extended to other features such as the arterial inflow behavior. The main factors distinguishing it from existing approaches are the data representation and task formulation, as well as a large dataset size with 500 liver lesions from two centers for algorithmic development and testing.

## 1 Introduction

Focal liver lesions are often detected as incidental findings during ultrasonic examinations of the abdomen. Although the standard B-mode images already show some distinct patterns, which are helpful for diagnosis, roughly 40% of lesions cannot be characterized considering this information alone.^1^ Magnetic resonance imaging (MRI) in combination with a contrast agent is the current gold standard for the diagnosis of liver lesions.^2^ In recent years, the use of contrast-enhanced ultrasound (CEUS) has evolved and gained increasing attention. A large multicenter study has proven the effectiveness of CEUS for diagnosis, especially that its accuracy is comparable to MRI.^3^ Ultrasound contrast agents consist of flexible microbubbles. When insonified with an ultrasound (US) pulse of intermediate power, they emit sound waves at frequencies different from the transmitted one, so called harmonic frequencies. Frequency filters can then be used to separate tissue from the agent signal.

Due to the dual blood supply system of the liver, three phases of blood and therefore contrast agent distribution can be differentiated with respect to the contrast agent injection: arterial (5 to 25 s), portal (25 to 60 s), and late phase (after 120 s). In each phase, the distribution characteristics of the agent provide relevant insights. Guidelines^4^ and decision trees^5^ are published, describing the connections between features visible in CEUS acquisitions and diagnosis. Unlike contrast to MRI, US contrast agents can be used for patients with renal insufficiency and show almost no adverse effects.^6^ CEUS acquisitions are fast and real-time and allow use of the same US-modality as the one where a lesion was detected initially. However, interpretation of CEUS sequences can be challenging due to the nonstandardized view and the high heterogeneity caused by motion (e.g., respiratory motion). In addition, the different contrast phases are typically acquired with intervals between them to prevent destruction of the microbubbles during prolonged imaging. This has the potential to further add to data heterogeneity when not using standardized acquisition times. Consequently, supporting clinicians in their diagnostic decision by means of an automated classification algorithm could help to expand the application range of CEUS in clinical praxis.

### 1.1 Related Work

Current studies to classify liver lesions either from B-mode or CEUS are collected in a recent review article.^7^ They can be categorized by their approaches to different aspects:

#### 1.1.1 Task formulation

Differentiating between malignant and benign lesions is the aim of the majority of studies.^8^^–^^16^ Classifying different lesion subtypes is less common and few studies attempt to discriminate between more than two subtypes.^17^^,^^18^ Several studies investigated algorithms to separate atypical focal nodular hyperplasia (FNH) from hepatocellular carcinoma (HCC), benign and malignant lesion types, which are especially difficult to distinguish.^19^^–^^22^

#### 1.1.2 Algorithmic approach

A typical approach considered in the majority of studies is to first extract features from selected frames or regions of interest (ROI) and use these features subsequently to train a machine learning classifier. Popular features are those extracted from time intensity curves (TICs)^9^^,^^10^^,^^18^ or a combination of TIC and morphological features.^13^^,^^16^^,^^17^ Features can also be extracted solely from images^11^^,^^12^^,^^15^^,^^19^^,^^21^ or from other derived properties such as the results of a tracking algorithm.^20^ Recently, approaches using deep learning (DL) techniques have been investigated more intensively. Zhou et al.^22^ combined handcrafted features and those extracted from a 3D neural network as input to a machine learning classifier. An approach using an image classifier applied pretrained ResNets to four selected CEUS frames and fused their outputs using a fully connected layer.^14^

#### 1.1.3 Motion compensation

Motion, mainly due to the patient’s breathing, is one of the greatest challenges for the automated analysis of CEUS sequences. Transducer movement, e.g., when the physician searches the spot where the lesion is visible best, also contributes. Due to motion, the lesion can change its relative position from frame to frame and even exit the imaged region completely (out-of-frame motion). There are several strategies implemented in the literature to cope with motion. Two studies described that patients were asked to hold their breaths during the examination.^17^^,^^22^ However, this is only an option when the examination period is short. Manual intervention is possible, for example, by discarding frames with out-of-plane motion^9^ or by manually adjusting ROIs on each frame.^18^ Some studies used motion compensation algorithms based on template matching^8^^,^^10^ or correlation analysis.^13^^,^^16^ Motion effects can also be restricted using frames in a time window (e.g., of 2 s) around an annotated one.^20^ Similarly, several studies only used between three to four manually selected frames from the different contrast phases^11^^,^^12^^,^^14^^,^^15^^,^^21^ and discard the remaining frames.

#### 1.1.4 Manual annotations

All examined studies require at least a lesion ROI on a single frame to be provided to the algorithms.^9^^,^^10^^,^^16^^,^^17^^,^^19^^,^^20^^,^^22^ Some studies demanded selection of an informative frame and ROI delineation in one frame per contrast phase^11^^,^^12^^,^^14^^,^^15^^,^^21^ and a few even in each frame.^13^^,^^18^ Frame selection is often necessary to limit the influence of motion as described in Sec. 1.1.3. In addition, manual ROIs should support the algorithms by providing only the relevant parts of the images and solving the problem of lesion detection.

The investigated studies showed good to excellent performance with accuracy between 78%^16^ and 98%,^22^ showing the potential of automated analysis of CEUS sequences. Their results are mainly limited by the size of their datasets. The study with the smallest dataset includes only 22 cases,^8^ and the largest number available is 363.^14^ From 15 investigated studies, seven include less than 100,^8^^–^^12^^,^^16^^,^^20^ and only two have more than 300 cases.^14^^,^^19^ Cases are mostly from a single center and recorded with one ultrasound machine. A secondary effect of the small datasets available is that all studies besides one^14^ do not have a dedicated test set. They would rather use a train-validation split or employ cross-validation to report their results. The reported performance values therefore have the potential to be too optimistic, especially when network parameters were tuned on the validation set.

### 1.2 Objectives

The overall objective of the present work is to investigate DL-based algorithms to support clinicians in the interpretation of CEUS acquisitions of focal liver lesions. Starting from approaches already implemented, the goal is to systematically explore new aspects, focusing on the integration of DL-based algorithms, which have not been extensively researched yet. The classification task investigated is to categorize the image feature washout. Washout describes a decrease in contrast agent intensity in the portal or late phase of lesion tissue faster than in the surrounding liver parenchyma. It is known as the main feature to separate malignant from benign lesions;^5^ therefore, it has a high clinical relevance. A third category of “neither” was included in this work to account for lesions, which do not take up contrast agent, for example, cysts, and therefore, also show no washout. Classifying a diagnosis-relevant image feature instead of the diagnosis itself allows integration of a variety of different lesions into the training. A data representation is proposed which is oriented at time intensity curves but makes it possible to use an image-based DL classifier. The representation is agnostic to the number of frames used, the video length, the image resolution, and the lesion size.

Several strategies to cope with the motion present in CEUS acquisitions are explored, starting with frame selection strategies based on correlation up to using an Optical Flow–based motion compensation. In addition, the influence of using a perfusion model fit to smooth the time axis is investigated.

## 2 Material and Methods

### 2.1 Data

**Fig. 1:**
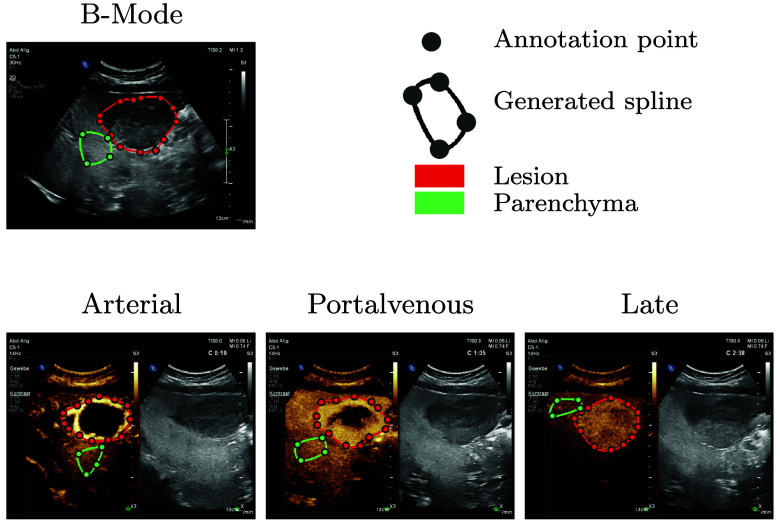
Annotated frames from one exemplary case, showing all available acquisitions (B-mode and three contrast phases). In each acquisition, the lesion as well as a parenchyma region is annotated by selecting points around the contour, which are automatically connected via spline interpolation.

Overall, 500 CEUS acquisitions of the liver from two centers (center one: 336, center two: 145) were collected between July 2022 and February 2024. The study was approved by the local institutional ethics committees (No. 19-705 and EA4/142/19) and informed consent was obtained from all patients. The imaging protocol includes B-mode US, color-coded duplex sonography, and CEUS in arterial, portalvenous, and late phase following the 2020 WFUMB guideline.^4^ The exact times at which a loop was recorded depended on the patient’s individual circulatory situation. The US examinations were performed using an Epiq Elite 7 (Koninklijke Philips N.V., Amsterdam, the Netherlands) in center one and an Aplio i800/i900 (Canon Medical Systems Corporation, Japan) in center two. SonoVue® (Bracco, Milan, Italy) was used as an ultrasound contrast agent. CEUS acquisitions were annotated by one radiologist per center using the software TOMTEC-ARENA (TOMTEC Imaging Systems GmbH, Unterschleißheim, Germany). In center one, the annotating radiologist was the same who also acquired the data, whereas in center two, acquisition and annotation were done by two separate radiologists. The annotators selected one representative frame from each phase and contoured the lesion as well as a parenchyma region. An exemplary case with its annotations is shown in Fig. 1. In addition, a structured reporting was filled out for each case, answering questions regarding the lesion type (diagnosis) as well as descriptive image features such as the washout characteristic. A total of 19 cases (3.8%) were excluded due to incomplete reporting or annotation, resulting in 481 liver lesions, which were eventually included in the analysis. Table 1 shows the different lesion types present in the dataset.

**Table 1:** Distribution of diagnoses in the collected dataset.

Diagnosis	Count
Hemangioma	136
Metastasis	93
FNH	74
Cyst	50
HCC	34
Abscess	17
Focal fatty deposition	16
Echinococcus cyst	15
Haemorrhagic cyst	12
Regenerated node	9
Focal fatty sparing	8
Other	7

### 2.2 Motion Compensation

Several approaches to compensate for the motion present in the acquisitions were investigated, including more than the three annotated frames in network training.

#### 2.2.1 Frame selection strategies

**Fig. 2:**
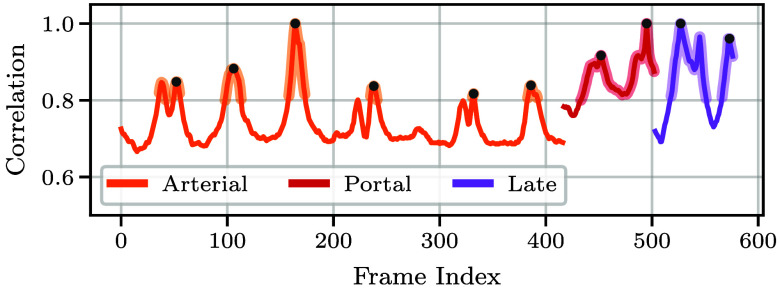
Exemplary Pearson’s correlation curve for one study to demonstrate the different frame selection strategies.

Frame selection strategies relied on the Pearson’s correlation between the annotated frame and all other frames in the respective series computed on the B-mode images. First, all frames above a defined correlation threshold were selected. Second, a peak-detection algorithm (find_peaks from scipy.signal^23^) was applied to extract regular peaks in the correlation curve, which are assumed to be related to regular breathing patterns. The algorithm is conditioned to find peaks which have a defined minimum height (similar to a correlation threshold), a minimum distance corresponding to roughly half a breathing cycle, and a prominence value defining how much the peak stands out from the baseline. Both strategies are exemplary visualized in Fig. 2. Several correlation thresholds were used for frame selection and subsequent training for both strategies. The annotation mask was applied to the additionally selected frames without any further adaption.

#### 2.2.2 Optical Flow tracking

**Fig. 3:**
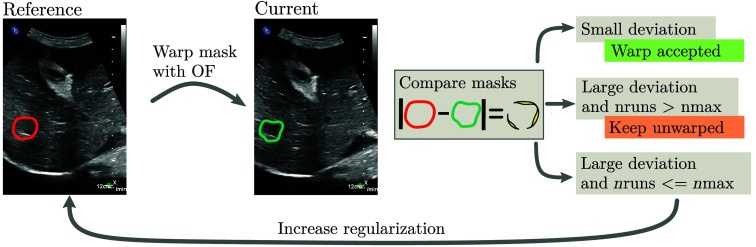
Strategy to find a reasonable parametrization for the Optical Flow algorithm per frame. Starting with a base parametrization, a displacement field is calculated between the reference frame and a current one and used to warp the annotation mask. Warped masks and reference masks are compared after aligning their centers of mass. In case their deviation is below a defined threshold, the warp is accepted. Otherwise, regularization is increased for the Optical Flow algorithm as long as either the deviation becomes acceptable or a defined number of maximum runs is reached. In the latter case, the reference image and mask are kept without warp.

When applying the unaltered annotation mask to the additionally selected frames, it will probably not cover the exact position of the lesion. In an effort to further reduce the effect of motion and improve the mask positioning, a motion compensation approach using Optical Flow was investigated. It utilizes the iterative Lucas–Kanade method to calculate displacement fields between the annotated reference frame and all remaining frames of the sequence for a set of sparse features. Therein, it is assumed that the motion inside a neighborhood can be described by an affine transformation, including translation, rotation, scaling, and shearing. Then, interpolation is used to derive displacement vectors for every pixel, which are used to warp the reference image and the annotation mask. To this means, calcOpticalFlowSparseToDense from the opencv library was used.^24^ For a large heterogeneous dataset in which motion occurs in various forms, it can be difficult to find a global OF parametrization, which leads to stable results in all cases. Therefore, an iterative scheme was established, as depicted in Fig. 3. Starting with a base parametrization ($\sigma_{\mathrm{OF}}=0.04$, $\lambda_{\mathrm{fgs}}=5000$, $\sigma_{\mathrm{fgs}}=3$), the OF field was computed between the reference and current frame and used to warp the annotation mask. Here, $\sigma_{\mathrm{OF}}$ is connected to the affine motion model and defines the influence of pixels from the neighborhood on the OF calculation depending on their distance to the current center pixel. Parameters $\lambda_{\mathrm{fgs}}$ and $\sigma_{\mathrm{fgs}}$ define a Gaussian postprocessing filter that controls the smoothness of the output field. Then, annotation and warped masks were compared by aligning their centers of mass and computing the difference between their areas. In case of a small deviation, defined as a difference, which is below 25% of the reference mask area, the parametrization was accepted, and the reference frame and mask were warped using the calculated displacement field. When the deviation was larger, the regularization parameter of the OF algorithm ($\lambda_{\mathrm{fgs}}$) was increased as long as either the deviation fell below the given threshold or a maximum number of repetitions (nmax=100) was reached. In the latter case, no motion compensation was applied, and the reference masks were used to identify the lesion and parenchyma areas on the current frame.

The results from the OF algorithm were used in two ways. First, the warped reference masks were used to identify the area of interest on frames, which were selected by the two frame selection strategies introduced above. Second, the Pearson’s correlation values for frame selection were computed using the warped reference frames instead of the static one. In case the motion compensation is successful, the correlation between the warped reference frame and the current frame is expected to increase. Therefore, a frame selection, which is based on such computed correlations, should extract frames where the warped mask fits the actual lesion position more adequately. Both possibilities are examined with both frame selection strategies and different correlation thresholds. In addition, to address observed variations in performance, models were trained in 21 independent runs (with the same configuration and data but random initialization), and evaluation metrics were averaged.

### 2.3 Data Representation

For any DL approach, the available data have to be converted into a representation in which it can serve as input to the desired classifier. In this work, an image-based classifier was used; therefore, the spatio-temporal data were converted into a 2D representation.

**Fig. 4:**
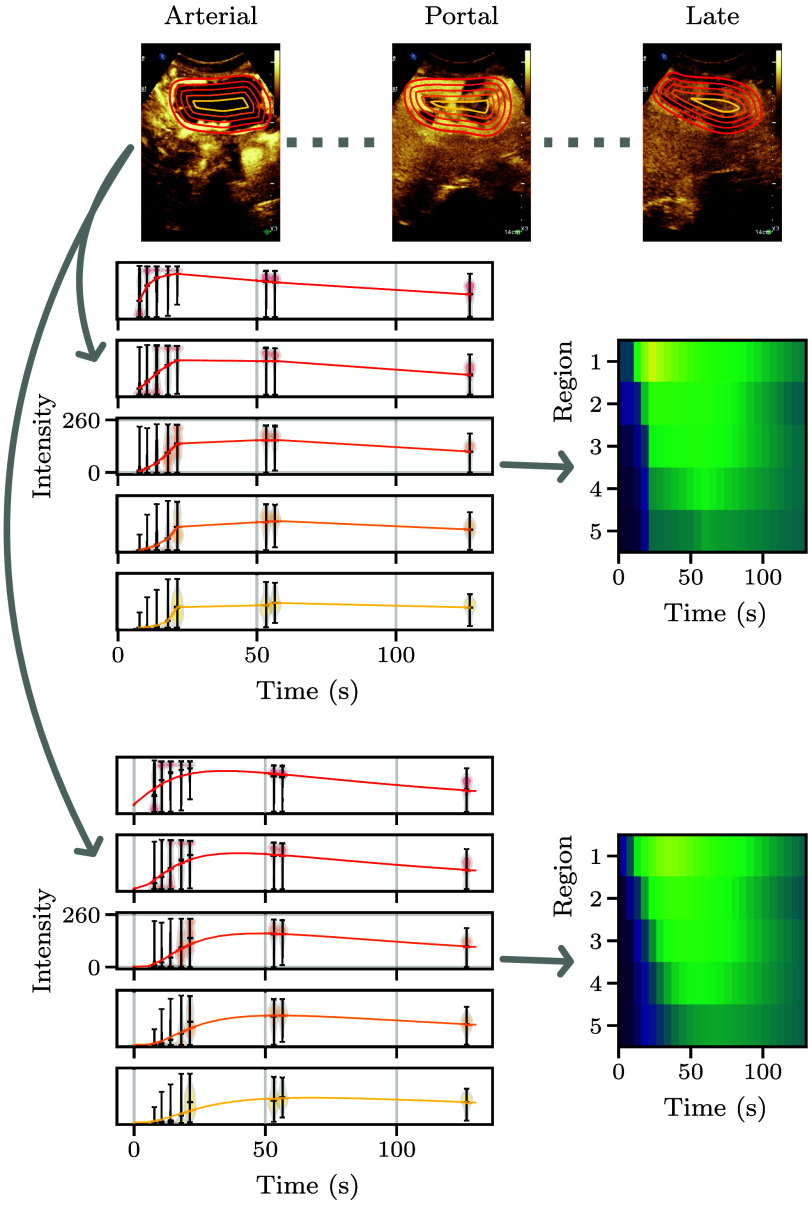
Construction of TIC maps using the annotation mask and additionally selected frames. The mask is divided into concentric regions. Values from each region build time intensity curves where each time point is represented by a distribution of values. Using a summarizing statistic such as the mean (shown in the middle part of the figure), a 2D representation is achieved where the x-axis denotes the time axis and the y-axis the respective region. Alternatively, a perfusion model fit can be used to get a representation of the contrast agent distribution (shown in the bottom part of the figure).

Inspired by time intensity curves, which are already used to quantify CEUS characteristics, those curves were extended to form so-called time intensity curve maps (TIC maps). The approach is visualized in Fig. 4: Starting from the three annotated frames, the lesion mask was distributed into concentric regions based on the distance of each pixel inside the mask to its contour. Therefore, the value range from zero to the maximum distance was equally binned so that the distance range covered in each region was the same. Overlap between the regions was allowed and specified as a percentage of the initial bin width. If the desired region count was not achieved with the initial overlap, the overlap was increased until it was met.

The lesion mask was applied to the extracted frames from the same sequence (In the example in Fig. 4, there are three additional frames for the arterial phase, one for the portal and no additional frames for the late phase). Then, TICs were constructed by combining corresponding regions from the annotation masks over selected frames. For each time point, where a frame is available, the TIC holds the distribution of values from the respective region, as can be seen in Fig. 4 at the lower left. The larger gaps between available time points show the specific noncontinuous acquisition characteristic of CEUS. By applying a summarizing statistic such as the mean, a single value per time point and region was extracted from each TIC and combined into a two-dimensional map (Fig. 4, middle part at the right). To get a continuous time axis from the often sparse and unevenly distributed observations, in the simplest approach, a linear interpolation was applied. A second possibility used was to fit a perfusion model to fill in missing values. This approach is described in detail in Sec. 2.4 and shown in Fig. 4 at the bottom.

### 2.4 Perfusion Curve Fitting

To obtain a linearly sampled time axis for the TIC maps, an alternative to linear interpolation is to fit a perfusion model to the data. This has the potential to model missing time points more reasonably. There are several different formulations of perfusion models used in the literature.^25^^,^^26^ Following a review article,^27^ the local density random walk (LDRW) was chosen as it was described to be suited best for modeling CEUS perfusion. A least-squares approach (using least_squares function from scipy.optimize^23^) was used to find a parametrization for the LDRW model based on the distribution of observed values from a specific region, e.g., the lesion, at selected time points. It was observed that the CEUS sequence acquired in the arterial phase was in the majority of cases longer compared with the portal and late phase. That is why, for most studies, there are more frames selected from the arterial phase than from the later phases. This implied that measurements from the arterial phase will have more impact on the perfusion curve fitting. To compensate for this effect, a linear weighting was applied to the squared residuals in the optimization, which accounts for the number of frames available per phase N by multiplying with $\frac{1}{N}$. Perfusion model fitting was deployed for both frame selection strategies with different correlation thresholds.

### 2.5 Deep Learning Training

As an image classifier, the ConvNeXt (Implementation available at PyTorch^28^) architecture was utilized, which is an adaption of ResNet using design choices adapted from vision transformers, for example, a larger kernelsize of 7×7^29^ and an inverted bottleneck structure. From the four variations with different depths and feature map counts, the *tiny* version was selected because it probably fits the limited amount of available training data best.

Data were split into training and test data with a ratio of 90% to 10%. The training set was further divided into three folds for cross-validation. Both splits were stratified with regard to the washout annotation. Class imbalance was observed as cases with “no washout” cover 53% of the entire data, whereas “washout” (28%) and “neither” (19%) cases are less common.

As input, TIC maps of both lesion and parenchyma concatenated in the channel dimension were used, and the classification layer of the network is adapted to three output classes {neither, no washout, washout}. TIC maps were constructed with 20 regions and 5% initial overlap for the lesion and one single region for the parenchyma, using the mean as a summarizing metric. The linear time axis of the TIC maps spans from 0 to 260 s with dt = 1 s, covering 98% of all late-phase acquisitions. Maps are resized to [224,260] to fit the expected input size of ConvNeXt in the first dimension but keeping the sample spacing of the time axis. To train the network, the AdamW optimizer^30^ with a learning rate $1\times 10^{-3}$, weight decay of 0.05, and cross-entropy loss was used. Training was performed for 600 epochs with a batch size of 10 for both training and validation. The model, which performed best regarding the validation loss, was selected as the final model. Two strategies to tackle the class imbalance were implemented. First, the training loss was weighted by the class percentages in each batch to avoid that the majority class dominates the loss. Second, an epoch sampling strategy was deployed in which data for one epoch is composed such that each class is equally represented.

## 3 Results

**Fig. 5:**
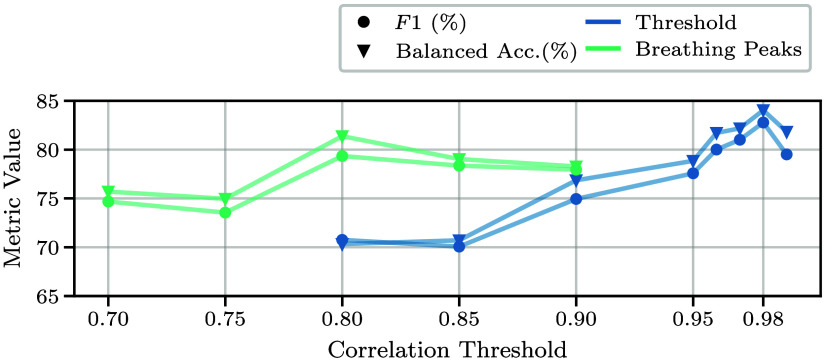
Performance metrics F1 and balanced accuracy on the cross-validation data using different frame selection strategies. Blue markers give the results for the correlation threshold frame selection, whereas the turquoise ones give those for breathing frame selection.

For the two frame selection strategies relying on a correlation analysis, the results in the form of F1 score and balanced accuracy are presented in Fig. 5. For the correlation threshold-based selection shown in blue, the worst result regarding the metrics is achieved using a threshold of 0.85 (F1=70.1%, ${\mathrm{acc}}_{b}=70.7\%$). Clear improvements can be accomplished by increasing the threshold, with the best result when using a value of 0.98 (F1=82.8%, ${\mathrm{acc}}_{b}=84.0\%$). For the strategy, which aims at extracting frames from the same breathing cycle, a minimum peak correlation of 0.80 results in the best performance (F1=79.3%, ${\mathrm{acc}}_{b}=81.4\%$), whereas higher or lower values lead to a degradation.

**Fig. 6:**
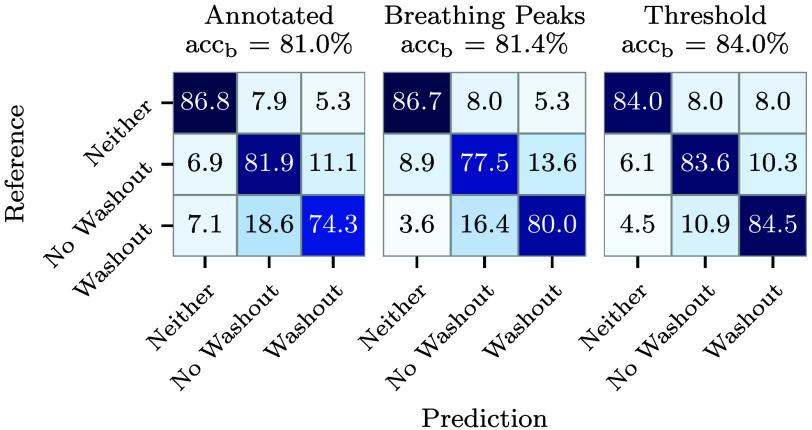
Confusion matrices for the three investigated frame selection strategies on the cross-validation data. Data are normalized per row and given in percent. The networks performing best for breathing and correlation threshold frame selection (thresholds 0.80 and 0.98) are shown.

To compare between a classification using only the three annotated frames as input and the best performing models from the frame selection strategies, corresponding confusion matrices are depicted in Fig. 6. Matrices are normalized per row, i.e., to the number of reference cases per class, implying that values on the diagonal directly correspond to the sensitivity for the respective class. The model trained on correlation-selected frames (with threshold = 0.98) achieves the best result and shows a similar sensitivity for all three classes. Although the other two approaches show better results regarding the “neither” class, using the annotated frames leads to worse performance especially on the “washout” cases. The breathing frame approach on the other hand classifies more “washout” cases correctly but also misses more “no washout” cases.

**Fig. 7:**
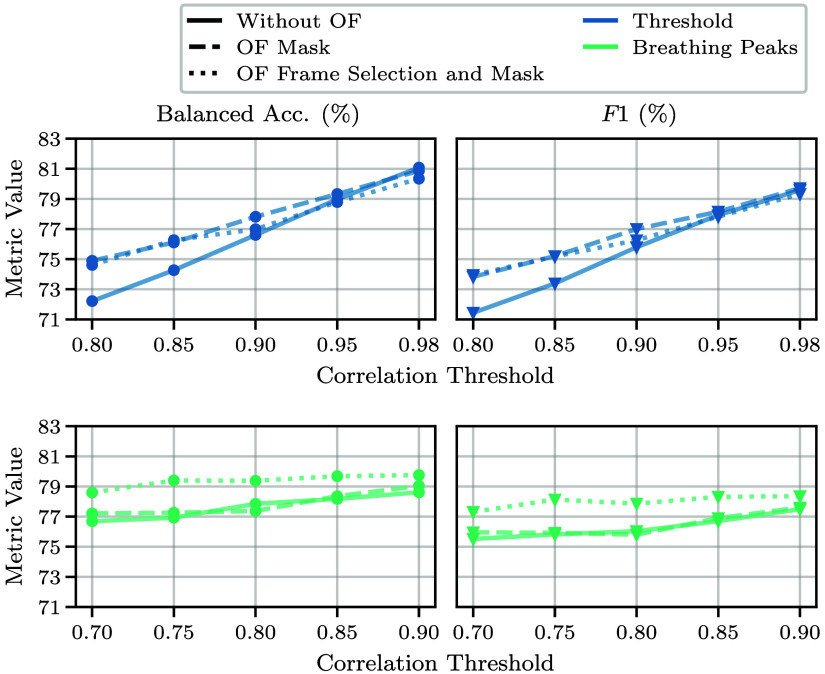
Comparison of Optical Flow approaches using either no compensation (“without OF”), the mask, which is warped using an Optical Flow field (“OF Mask”), or in addition to the warped mask also a frame selection, which is performed on the warped reference frames (“OF frame selection and mask”). The upper plots show results for the threshold-based frame selection and the lower plots those for the breathing peak approach. Metrics are averaged over 21 model runs.

A comparison of the two approaches using motion compensation with OF with the uncompensated case is shown in Fig. 7 for both correlation-based frame selection strategies (correlation threshold and breathing peak). Networks were trained with five different correlation thresholds for frame selection. It can be observed that including motion compensation increases the model performance, especially for lower correlation thresholds for both frame selection strategies. The variant, which uses a compensated mask as well as the warped reference frames for frame selection, works slightly better. Considering higher correlation values for the threshold-based approach (0.95 or 0.98), differences between the metrics become minimal, and for 0.98, the network without motion compensation achieves the best results. Considering the breathing peak approach, the approaches using motion compensation consistently achieve higher performance metrics for the investigated thresholds.

**Fig. 8:**
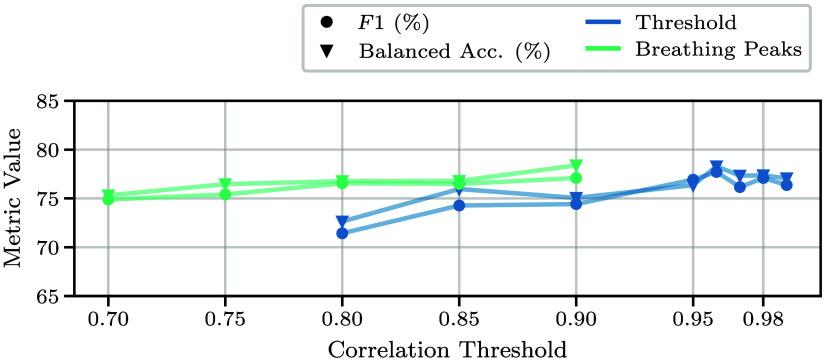
Performance metrics F1 and balanced accuracy on the cross-validation data using different frame selections and a perfusion curve fit.

For models trained with a perfusion curve fit, a comparison among different frame selection strategies is shown in Fig. 8. Trainings with frames selected via different correlation thresholds show a slight increase in performance, reaching the optimum at 0.96 (F1=77.7%, ${\mathrm{acc}}_{b}=78.3\%$). Using a breathing approach, the performance values are almost equal, and the balanced accuracy only slightly increases when using a threshold of 0.90 (F1=77.1%, ${\mathrm{acc}}_{b}=78.4\%$). In general, the performance of the networks using the perfusion fit decreases in comparison with their counterparts using linear interpolation.

**Fig. 9:**
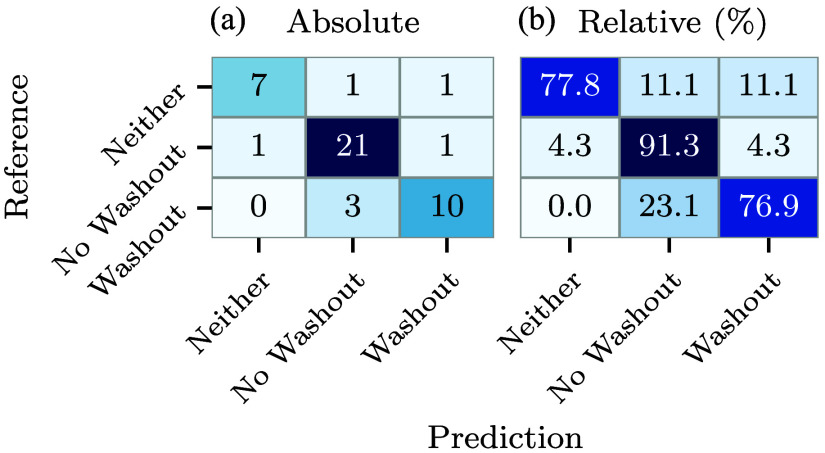
Confusion matrices when applying the best-performing model on the test data. (a) Absolute case numbers are displayed. (b) Relative values normalized per row.

The results for applying the best-performing model (frame selection with correlation threshold 0.98, linear interpolation) to the hold-out test data are depicted in Fig. 9. The balanced accuracy is at 82.0% with an F1 score of 83.4%.

## 4 Discussion

This work investigated different strategies to classify the image feature washout—which is an important separator between malignant and benign lesions—from CEUS acquisitions of the liver. The classification was approached by applying an image-based classifier to two-dimensional maps, which are oriented at time intensity curves. As the lesion was only annotated on one frame per acquired CEUS sequence, one aspect investigated was how to best select additional frames from the sequences and whether motion compensation is beneficial. In addition, the impact of a perfusion curve fit in comparison to linearly interpolating missing time points was evaluated.

### 4.1 Frame Selection and Motion Compensation

Washout classification already leads to reasonable results when using the three annotated frames only (${\mathrm{acc}}_{b}=81.0\%$, see Fig. 6). However, adding more information by selecting additional frames, which are above a given correlation threshold, can lead to improvements. This is the case for thresholds from 0.96 to 0.98 with the best result achieved using 0.98 (${\mathrm{acc}}_{b}=84.0\%$). Lower thresholds did not show an improvement compared with using the annotated frames only, indicating that additionally selected frames need to have a high similarity to the annotated one, probably to avoid including parenchyma tissue into the lesion map. Using a selection of frames from the same breathing cycle by searching for peaks in the correlation curve had the intention to extract information along the whole timeline of the sequences. Here, performance decreased when using higher correlation thresholds for peak detection probably because the number of additionally selected frames drops toward zero. As this approach did not show better results compared with the frame selection using a simple correlation threshold, it seems like extracting frames with similar content is more important than achieving a dense sampling of the time axis.

The results of the frame selection investigation imply that a high similarity in selected frames is important for the classification. Hence, the idea to apply additional motion compensation to adapt the annotation mask seems to be reasonable. Using a variation of the OF algorithm for both frame selection as well as warping the annotation mask improved the results (see Fig. 7). The observed trend that the influence of motion compensation declines when using a higher correlation threshold for frame selection is reasonable as the more similar the content of the selected frames gets, the less difference will be detectable between warped and nonwarped annotation mask. The performance however did not exceed the best one achieved without motion compensation, at least for the threshold-based frame selection approach. One possible reason for this observation could be that the approach using OF is not well suited for the given kind of tracking task on CEUS acquisitions. Motion compensation on ultrasound liver data is quite challenging due to through-plane motion, caused by either patient breathing, coughing, or transducer motion. OF however is based on the assumption of brightness consistency, which means that objects are assumed to move only in-plane and keep their overall intensity. This assumption is certainly not met in the presence of through-plane motion. Acquisitions from different patients show motion of varying extents and manifestations. Therefore, parametrization of the OF algorithm had to be adapted per acquisition. The criteria used here were based on the similarity between the annotation mask and its warped version (see Fig. 3). Similar to the brightness assumption of OF, this restricts the application to in-plane motion as only displacement of the mask and small shape deviations are allowed.

It is possible that more advanced motion compensation techniques could lead to better results in terms of frame selection and mask warping. However, the question whether an improved motion compensation technique would at the same time improve classification performance can not be answered clearly. It is not obvious that the wrongly classified cases—from which some will be discussed in Sec. 4.3—would improve when more information along the time axis would be available.

### 4.2 Perfusion Modelling

**Fig. 10:**
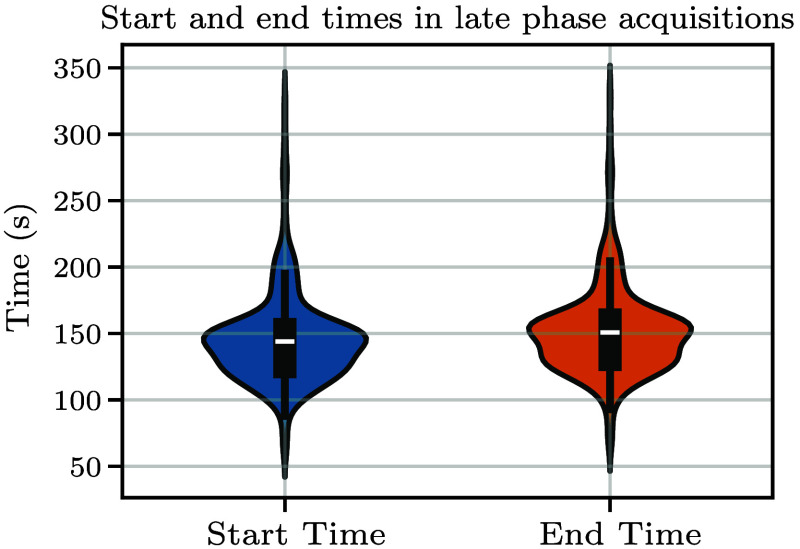
Distribution of start and end times for the late phase acquisitions.

For the presented approach, using a perfusion curve fit for the TIC maps did not lead to better results compared with linear interpolation. One explanation could be that fitting of the LDRW model can only be performed reliably with a large number of measurements as there are commonly available in corresponding literature^25^^,^^26^ but not necessarily in the presented approach when using high correlation thresholds. However, as the performance tends to improve with increasing correlation threshold, using less frames for curve fitting does not seem to be responsible for the performance difference. A different hypothesis is based on the observation that the start and end times of the late phase acquisitions show a large spread in the available data set, as shown in Fig. 10. For constructing TIC maps, all studies are projected to one common time axis, and therefore, extrapolation will take place for all studies where the last selected frame occurs earlier than the endpoint of the common time axis. In the presented approach, this endpoint was set to t=260  s, implying that roughly 97% of studies are affected by extrapolation. There are fewer frames in the late phase, which makes the perfusion fit already less reliable there, although it was tried to mitigate this aspect using a weighing factor for the least-squares fit. Using the LDRW model for extrapolation could introduce errors in the sense that the curve does not represent the behaviour of the contrast agent. When using linear interpolation, extrapolation just means retaining the last observed value. This does not fit the contrast agent behaviour either. However, as physicians selected the late phase sequence from a potential pool of acquisitions with the criterion that they can see the lesion and the washout information best, linearly extrapolating preserves the clinically important information. To avoid extrapolation, the endpoint of the common time axis might be set to a lower value. This would exclude late-phase information for those studies with start times above the chosen end time. From a clinical perspective, the late phase is particularly important to assess the washout characteristic. In this trade-off between avoiding extrapolation and including as much information from the late phase as possible, more priority was placed on the latter.

### 4.3 Investigating Challenging Cases

**Fig. 11:**
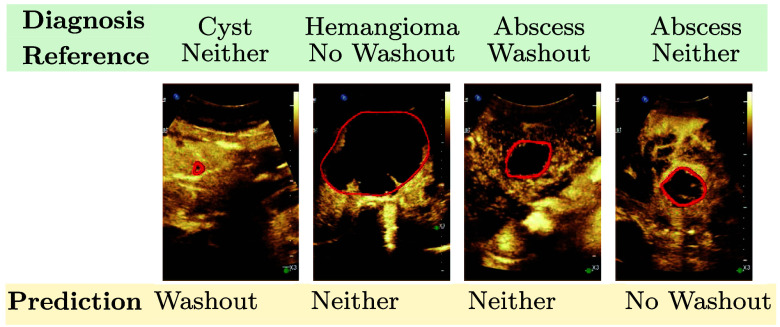
Four exemplary wrongly classified cases. For each study, the annotated frame from the arterial phase is shown with diagnosis and reference washout given above and predictions from the best-performing classifier below the images.

Having a look at cases from the validation set, which are wrongly classified using the best available model, some possible causes for misclassification can be identified, as visualized in Fig. 11. First, very small lesions can be difficult for the classifier. The exemplary cyst shown at the outer left in Fig. 11 is not taking up contrast agent and therefore has been assigned the reference label “neither.” The reference annotation does not exactly match the border of the lesion and also includes some enhancing parenchyma, probably due to the small lesion size. This can serve as an explanation as to why the case is wrongly classified as “washout.” In the validation set, five of those very small lesions could be identified (roughly 8% of the wrongly classified cases). A second observation is that hemangiomas, which show contrast intake mainly at the vicinity of the lesion border, are sometimes predicted to show “neither” instead of “no washout.” This is often the case for very large hemangiomas, as depicted in Fig. 11, in the second image from the left and make up 17% of the wrongly classified cases. Here, the enhancement is only minimal, and the inner part, which does not take in contrast agent, dominates the TIC map such that the behavior can resemble that of a cyst without contrast intake. Third, abscesses have shown to be difficult due to heterogeneous reference labels. From the 14 abscesses, nine are referenced to show “neither,” three “no washout,” and two “washout.” Two examples are shown on the right side of Fig. 11. The first abscess appears nonenhancing; therefore, the predicted label “neither” seems reasonable despite the fact that the reference label is “washout.” For the second abscess, on the other hand, “neither” would meet the reference label but probably due to enhancing structures visible inside the lesion, the classifier does not come to this result. The heterogeneity in the reference label could be caused by the fact that abscesses often show rim enhancement, in which case the reference label could relate to this enhancement and not to the general contrast intake of the lesion, depending on which one the radiologist deems to be more important. In addition, in some cases, for example, the one on the very right of Fig. 11, the ultrasound imaging plane includes the outer part of the lesion where already some “background enhancement” is visible. This enhancement does not take place inside the cyst but is just visualized there due to the non-zero US slice thickness. This makes it difficult for the classifier to match the clinical reference.

#### 4.4 Comparison to Literature Approaches

**Fig. 12:**
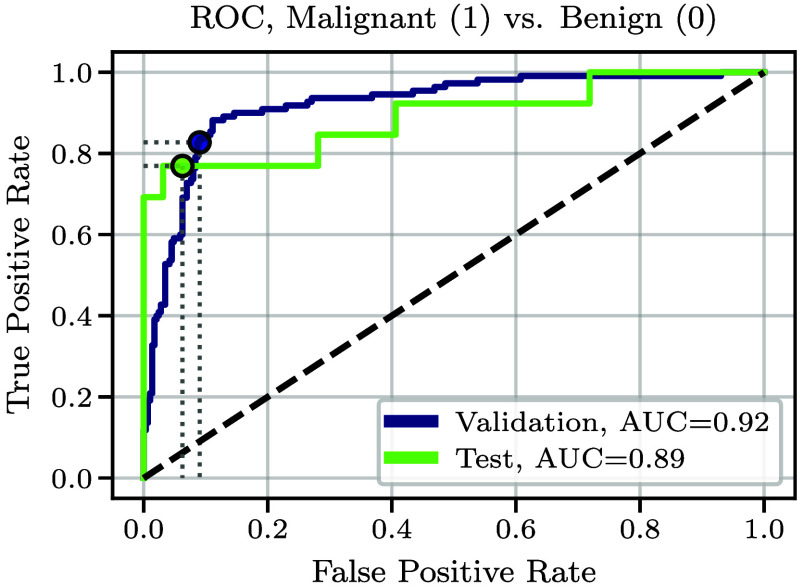
Receiver operating characteristic for the best classifier when reformulating the washout classification as a 2-class problem. The operating points for a threshold of 0.5 are shown, above which cases are classified as being malignant.

In the literature, no dedicated study that aims to specifically classify washout could be found. Nevertheless, as many studies focused on differentiating benign from malignant lesions, those can be used for comparison. Therefore, the two classes (and their scores) “neither” and “no washout” were combined into a class holding benign cases, whereas the “washout” class is equivalent to the malignant label. Performances of classifiers found in the literature rank between accuracies of 78.0% (sensitivity = 76.0%, specificity = 92.0%)^16^ and 91.0% (sensitivity = 92.7%, specificity = 85.1%).^14^ With an operating point of 0.5, which is equivalent to select the class with the maximum score, the best washout classifier reaches an accuracy of 89.0% (sensitivity = 82.7%, specificity = 91.3%) on the cross-validation data and 88.9% (sensitivity = 76.9%, specificity = 93.4%) on the test data. A receiver operating characteristic (ROC) is given in Fig. 12. The presented model therefore seems to fit into the landscape of already conducted studies and although it does not outperform the best ones, it is closer to the best-performing than to the worst-performing models. Generally, comparison with literature studies is challenging due to their high variability in study design. In addition, most investigations include a relatively low number of lesions (below 100 cases), which is improved upon in this work with 481 available studies.

### 4.5 Limitations and Further Directions

Thinking about the clinical application scenario of the presented approaches, one limitation is that there needs to be manual input in the form of a lesion and parenchyma annotation in one selected frame of each contrast phase. However, CEUS acquisitions are only performed when there is a suspicious lesion visible in the B-mode image, so at acquisition time, the clinician already knows where the lesion is. Detecting the lesion in CEUS is then considered not to be the main challenge, but assessing the contrast behaviour and the related diagnosis correctly is. An automated detection algorithm would save clinicians time, but it could be more important to support them in their diagnostic decision than to solve a task, which is not difficult for them. Manual effort could be tried to be reduced by requiring only one annotation in the arterial phase. It then has to be investigated whether the annotation can be reliably extended to the other phases. The answer probably depends on how much motion—either from the patient or by replacing the transducer—is present between the acquisition of the different phases.

Looking at the algorithmic performances and the discussed wrongly classified cases, there is room for improvement and future work. Reference annotations for the heterogeneously labeled abscess cases should be clarified and adapted. Each case was assessed and annotated by only one radiologist from the institution where data were also acquired. Therefore, it can not be ruled out that information other than the CEUS sequence, e.g., the anamnesis of the patient, influenced the washout assessment. An interrater study to investigate whether different radiologists perceive image features in CEUS similarly would be of great interest. The size of the lesion has been shown to be a factor for wrong classifications, for example, for tiny lesions or for large hemangiomas. It is probably difficult to include the size of the lesion directly into a neural network architecture, but it could be included in the data preprocessing. For small lesions, frame selection could be restricted, e.g., by computing the correlation only in a patch around the lesion or by adapting the correlation threshold with the lesion size. For very large lesions, the annotation mask could be enlarged a little bit to make sure that border enhancement is sufficiently included. This on the other hand brings the risk of including parenchyma and misclassifying, for example, cysts. Such forms of data augmentation would also increase the number of available training samples and could contribute to making the training more robust. Different network architectures for the image classifier could be investigated as well as different settings for the TIC map creation. Testing the algorithm on datasets from different centers would be favourable to evaluate the robustness of the approach regarding different US scanners and operators.

## 5 Conclusion

This work presented a DL-based approach to classify the diagnosis-relevant image feature washout from CEUS acquisitions of focal liver lesions. Important aspects such as motion compensation and the possible benefits of using a perfusion model were investigated. The classification showed promising results with a maximum balanced accuracy of 84.0% on the validation and 82.0% on the test data. Frame selection with a high correlation threshold performed best, implying that the information searched for can already be retrieved from a few selected frames. Neither additional motion compensation using Optical Flow nor perfusion curve fitting leads to improved performance.

Classifying important image characteristics could be extended beyond washout, e.g., to the inflow pattern in the arterial phase, to provide more diagnostic distinction besides categories malignant and benign. Such approaches introduce some sort of interpretability as the clinical users can compare their own perception of the acquisition with the classifier output.

## Data Availability

Data are not publicly available due to data protection and privacy rules.
